# Pennsylvania Hospital

**Published:** 1849-04

**Authors:** Spenser Sergeant

**Affiliations:** Resident Physician; Pennsylvania Hospital


### PENNSYLVANIA HOSPITAL.—*Surgical Wards.—Service of* DR. PEACE.

### *Cases discharged since February 15th*, 1849.

| | Cured. | By request. | Died. |
|---|---|---|---|
| Abscess, - - - - - | 1 | 0 | 0 |
| Concussion of Brain, - - | 1 | 0 | 0 |
| Contusions, - - - - | 5 | 0 | 0 |
| Dislocation, - - - - | 1 | 0 | 0 |
| Erysipelas, - - - - - | 1 | 0 | 0 |
| Fractures, simple 10, viz.: | | | |
| Nose, - - - - - | 1 | 0 | 0 |
| Ribs, - - - - - - | 0 | 1 | 0 |
| Arm, - - - - - - | 3 | 0 | 0 |
| Fore-arm, - - - - | 1 | 0 | 0 |
| Leg, - - - - - - | 1 | 1 | 0 |
| Patella, - - - - - | 1 | 0 | 0 |
| Pelvis, - - - - - - | 1 | 0 | 0 |

| | Cured. | By request. | Died. |
|---|---|---|---|
| Fractures, compound 1, viz.: | | | |
| Skull, - - - - - - | 1 | 0 | 0 |
| Fistula-in-ano, - - - - - | 0 | 1 | 0 |
| Gonorrhœa, - - - - - | 1 | 0 | 0 |
| Hernia, humoralis, - - - | 1 | 0 | 0 |
| Inflamed gland, - - - | 1 | 0 | 0 |
| " leg, - - - - - | 1 | 0 | 0 |
| Iritis, (syphilitic,) - - - | 1 | 0 | 0 |
| Ophthalmia, - - - - - | 1 | 0 | 0 |
| Paronychia, - - - | 1 | 0 | 0 |
| Retention of urine, - - - | 2 | 0 | 0 |
| Syphilis, - - - - - | 6 | 0 | 0 |
| Tumor, - - - - - - | 1 | 0 | 0 |
| Wounds 9, viz.: | | | |
| Incised, - - - - - - | 2 | 0 | 0 |
| Lacerated, - - - - - | 3 | 0 | 1 |
| Gunshot, - - - - - | 4 | 0 | 0 |
| Ulcers, - - - - - - | 6 | 0 | 1* |
| | 49 | 3 | 1 |

The following, among the cases discharged, are of sufficient interest to give some of the details:

*Fracture of the Pelvis—Rupture of the Urethra—Fistula in the Groin—Recovery.*—O. Murray, Irish, æt. 23, was admitted Nov. 23d, 1848, and states that about three weeks before, he was injured by the falling of a bank of earth, the greatest weight of which fell across his pelvis and thighs. He was unable to rise, or, when raised, to support himself on his legs. The following day, he discovered that he was unable to pass any urine, and three days were allowed to elapse before the catheter was employed. It was not retained in the bladder, but after its introduction he could, though with great pain and difficulty, relieve himself. In the course of the ensuing week, the scrotum became very much swollen and inflamed, and a superficial slough separated from the left groin, which had been the most contused. Very soon afterwards the urine was observed escaping through an opening in the ulcer in the groin, and the practitioner attempted unsuccessfully to pass a catheter into the bladder. The next day the patient was brought to the Hospital.

When admitted, he was very much emaciated, and his back, in several places ulcerated from pressure.

In the left groin there was an unhealthy looking ulcer, three or

* Died of gangrene of the leg.

four inches in circumference, through a small opening in which the urine constantly escaped. Urine also was infiltrated down the thigh, and mixed with air and pus, could be seen escaping from the ulcer above, when pressure with the hand was made from the knee up. Upon examining the pubic arch, crepitus could be felt very distinctly. The fragments were very moveable, and the fracture was thought to be comminuted. There were no other injuries.

A gum elastic catheter was with some difficulty passed into the bladder and retained there, and to prevent any further infiltration in the thigh, a roller was applied from the toes up, and the limb elevated. A poultice was applied to the ulcer, and the patient was ordered a generous diet, with porter.

On account of the extensive laceration of the urethra, the urine did not all escape by the catheter, but, mixed with pus, occasionally made its way through the ulcer in the groin. This, however, gradually ceased, and the ulcer commenced cicatrizing.

About the beginning of his second week in the Hospital, and as the ulcer in the groin commenced cicatrizing, a urinary abscess formed in the perineum; this opened, discharged for about a week, and then healed. The catheter was changed every second day.

In the meantime the patient's general health rapidly improved, and the fracture became consolidated.

Feb. 12th, 1849. To-day the ulcer is entirely healed, and patient was allowed to sit up.

Feb. 18th. Withdrew the catheter which he has worn constantly since in the hospital. He passes his water without any difficulty whatever. The fracture is perfectly firm, and a considerable quantity of new bone can be felt in the vicinity. Walks very well. Discharged well, March 8th, 1849.

*Compound fracture of the Skull—Wound of Dura-Mater—Recovery.*—Martha Moore, æt. 18, coloured, admitted December 31st, 1849, with an injury of the head, caused by a blow from a hatchet.

There was an external wound about 2½ inches in length in the forehead, near the junction of the sagittal and coronal sutures, and a fracture could be felt the whole extent of the wound, the edges somewhat depressed and comminuted. The patient stated that immediately after the receipt of the blow, she was, for a short time, insensible, and afterwards sick at the stomach; but when admitted, she presented none of the symptoms which usually attend so serious an injury. She walked some distance to the hospital; she had no marked stupidity, and the pupils contracted naturally. She had lost scarcely any blood, but her pulse was frequent and feeble.

She was put to bed; the head shaved and covered with cold cloths, and wound closed with adhesive plaster. Ordered calomel, gr. x., to be followed by salts, and barley-water for diet.

On removing the dressings the third day after her admission, the wound was found suppurating freely, and with the pus were small portions of the cerebral substance, showing that the dura mater was wounded. She had, however, no bad symptoms; her skin was cool, her pulse had fallen to 80, she had scarcely any pain in her head, and her intelligence was perfect. She remained without change until the 18th of Jan., 11 days after her accident, when the discharge of cerebral substance ceased, though the pulsations of the brain were visible, the discharge welling out at each pulsation. Pulse 68. No pain in head or other bad symptoms. Has continued the same diet of barley water and gruel, and occasionally purged with salts.

By the 20th the wound had nearly cicatrized and the pulsations of the brain were no longer to be seen.

Feb. 5th. Wound entirely healed, and patient allowed to arise. She remained without a bad symptom, and Feb. 18th she was discharged well.

*Gun shot wound of the fore-arm—Recovery.*—A boy aged 14 was admitted Jan. 29, 1849, with a singular wound of the fore-arm. Two pistol bullets had passed directly through the arm, entering on the palmar surface of the lower third, without wounding either of the bones or the main vessels of the limb. There was no hemorrhage, the radial and ulnar arteries could be felt beating below, and the hand could be pronated and supinated with very little pain. There was considerable inflammation of the arm for a few days, and small sloughs separated from the wounds of entrance and exit.

The arm was laid on a splint and covered with a poultice.

He was discharged well, all the motions of the arm perfect, Feb. 20th.

*A stab of the chest—Recovery.*—A policeman, æt. 44, was admitted Feb. 2d, 1849 with a stab of the upper part of the right chest. He was said to have lost a large quantity of blood. His pulse was very feeble and frequent. Respiration 32, and difficult. He had no cough or bloody expectoration. The wound was closed with adhesive plaster and dry lint, and the patient's head and shoulders elevated on a bed chair. He was too feeble to examine posteriorly, but in front on the injured part the sound on percussion was good, and the respiratory murmur could be heard throughout. There was considerable emphysema of the subcutaneous cellular tissue. Ten hours after admission he had reacted. His pulse became 94, and full. Respiration 44, and more difficult. Ordered V. S. until the pulse fell, and calomel gr. ss., Dover's powd. gr. v., every six hours. Ten ounces of blood were taken from the arm, when the dyspnœa decreased considerably, and there was copious perspiration.

5th. Has continued improving. No fever or dyspnœa. In front the respiratory murmur is heard throughout, and the percussion is good. Behind, which was examined for the first time, perfect flatness on percussion, and tubular respiration over the whole lung except at the summit, where the vesicular murmur is heard feebly. Suspended calomel and Dover's powd., as mouth is slightly touched. Continue gruel diet.

By the 8th the wound had healed, but the auscultatory signs were the same.

13th. The tubular respiration of the injured side behind giving way; vesicular murmur heard feebly mixed with subcrepitant rale; still comparative dulness on percussion. Allowed soup diet with milk. By the 18th, the respiratory murmur could be heard feebly throughout the right lung, behind, and the sound on percussion was much clearer. Allowed to arise.

23d, discharged well. SPENSER SERGEANT, M. D.
*Resident Physician.*

*Pennsylvania Hospital, March,* 1849.

